# Torsade de pointes: A nested case–control study in an integrated healthcare delivery system

**DOI:** 10.1111/anec.12888

**Published:** 2021-09-21

**Authors:** Neha Mantri, Meng Lu, Jonathan G. Zaroff, Neil Risch, Thomas Hoffmann, Akinyemi Oni‐Orisan, Catherine Lee, Carlos Iribarren

**Affiliations:** ^1^ Department of Cardiology Kaiser Permanente San Francisco Medical Center San Francisco CA USA; ^2^ Division of Research Kaiser Permanente Oakland CA USA; ^3^ Institute for Human Genetics University of California, San Francisco San Francisco CA USA; ^4^ Department of Epidemiology and Biostatistics University of California, San Francisco San Francisco CA USA

**Keywords:** case, control study, long QT syndrome, risk factors, torsade de pointes

## Abstract

**Background:**

TdP is a form of polymorphic ventricular tachycardia which develops in the setting of a prolonged QT interval. There are limited data describing risk factors, treatment, and outcomes of this potentially fatal arrhythmia.

**Objective:**

Our goals were as follows: (1) to validate cases presenting with Torsade de Pointes (TdP), (2) to identify modifiable risk factors, and (3) to describe the management strategies used for TdP and its prognosis in a real‐world healthcare setting.

**Methods:**

Case–control study (with 2:1 matching on age, sex, and race/ethnicity) nested within the Genetic Epidemiology Research on Aging (GERA) cohort. Follow‐up of the cohort for case ascertainment was between January 01, 2005 and December 31, 2018.

**Results:**

A total of 56 cases of TdP were confirmed (incidence rate = 3.6 per 100,000 persons/years). The average (SD) age of the TdP cases was 74 (13) years, 55 percent were female, and 16 percent were non‐white. The independent predictors of TdP were potassium concentration <3.6 mEq/L (OR = 10.6), prior history of atrial fibrillation/flutter (OR = 6.2), QTc >480 ms (OR = 4.4) and prior history of coronary artery disease (OR = 2.6). Exposure to furosemide and amiodarone was significantly greater in cases than in controls. The most common treatment for TdP was IV magnesium (78.6%) and IV potassium repletion (73.2%). The in‐hospital and 1‐year mortality rates for TdP cases were 10.7% and 25.0% percent, respectively.

**Conclusions:**

These findings may inform quantitative multivariate risk indices for the prediction of TdP and could guide practitioners on which patients may qualify for continuous ECG monitoring and/or electrolyte replacement therapy.

## 1 INTRODUCTION

Torsade de Pointes (TdP), first described in 1966,(La, 1966) is a potentially life‐threatening polymorphic ventricular tachycardia associated with prolongation of the QT interval on the 12‐lead electrocardiogram (ECG).(Fontaine et al., 1982; Gupta et al., 2007) Because TdP is a rare diagnosis without specific *International Classification of Diseases* (ICD)‐9 or ICD‐10 codes, there are limited data describing clinical risk factors and management strategies for this malignant arrhythmia.(Kay et al., 1983).

The established risk factors for TdP include older age, female sex, heart disease, electrolyte disorders (especially hypokalemia and hypomagnesemia), renal or hepatic dysfunction, bradycardia or rhythms with long pauses, treatment with more than 1 QT‐prolonging drug, and genetic predisposition.(Drew et al., 2010; Faber et al., 1994).

The goal of this study was twofold: first, to validate a case series of patients presenting with TdP and compare these patients to age‐, sex‐, and race/ethnicity‐ 2:1 matched controls in order to identify modifiable risk factors and secondly, to describe the management strategies used for TdP and the short‐term survival and long‐term survival in a real‐world healthcare setting.

## 2 MATERIALS AND METHODS

### 2.1 Population and study design

Kaiser Permanente Medical Care Program (KPMCP) of Northern California is an integrated healthcare delivery system serving approximately 4.5 million members. The membership is stable with less than 10% turnover annually overall and <3%–5% among members 65 years of age and older and/or who have a chronic condition. The program delivers comprehensive inpatient and outpatient care to its members and captures many aspects of its care using its multiple comprehensive clinical and administrative databases. Kaiser's population is ethnically and socio‐economically diverse and is broadly representative of the Northern California population.(Krieger, 1992).

Our study was based on the GERA (Genetic Epidemiology Research on Aging) cohort. The goal of the GERA cohort was to create a large, multiethnic, and comprehensive population‐based resource into the genetic and environmental basis of common age‐related diseases and their treatment, and factors influencing healthy aging and longevity.(Banda et al., 2015; Kvale et al., 2015) The GERA cohort consists of a diverse cohort of 110,266 members of the Kaiser Permanente Medical Care Plan, Northern California Region (KPNC) aged 18 and older, who participated in the KPNC Research Program on Genes, Environment, and Health (RPGEH) and provided a saliva sample for DNA analyses. The RPGEH utilizes the longitudinal electronic health records (EHR) of KPNC to obtain clinical, laboratory, imaging, and pharmacy information on all cohort members, to which personal demographic, behavioral, and health characteristics have been added through member surveys.

We used *ICD*‐*9* code 427.1 and *ICD*‐*10* code I47.2, augmented with natural language processing (NLP), to identify all cohort members who developed paroxysmal ventricular tachycardia between January 1, 2005, and December 31, 2018. We identified a total of 2,528 subjects with a code for paroxysmal ventricular tachycardia, and, among them, 132 had a mention of “torsade” or “torsades” in the medical record. Manual chart review, including examination of 12‐lead ECGs, telemetry strips, ambulatory ECGs, and medical documentation, was performed for these 132 patients to confirm “true” TdP events. The final cohort of patients with a confirmed TdP event by a cardiologist (N.M.) comprised 56 persons. The 76 patients without validated TdP events typically mentioned "torsade" within the differential diagnosis given baseline risk factors for TdP (prolonged baseline QTc, electrolyte abnormalities, use of multiple QT‐prolonging pharmacotherapies) or documented possible "torsade" based on inpatient telemetry monitoring. However, none of these patients had a confirmed "torsade" event documented in the electronic medical record by a physician. A second physician investigator (C.I.) reviewed 25 randomly selected eligible cases for review, and agreement with the first adjudicator was 100% (12 true cases out of 25). Thus, our code plus NLP algorithm to identify and validate TdP had a positive predictive value of 42% (56/132). Each of these TdP patients was matched with 2 controls with the same birth year, sex, and race/ethnicity. Moreover, control subjects were assigned an “index” date which was the hospitalization date of the corresponding matched case.

In both cases and controls, gender, date of birth, and race/ethnicity were obtained from the patient's demographic file. Diabetes was ascertained through linkage with the KPNC Diabetes Registry.(Karter, 2006) The prevalence of other comorbidities, laboratory values, body mass index (BMI), and smoking status was extracted from the EHR. A list of *ICD*‐*9*, *ICD*‐*10*, and current procedural terminology, fourth version (*CPT4*) codes is provided in (online‐only) Appendix SA. Baseline QT_c_ interval (with Bazett correction), available from a Regional ECG database, was from the most recent 12‐lead ECG prior to index admission for TdP or assigned index date for controls, going back up to 5 years. Event QT_c_ (only in cases) was determined from the baseline 12‐lead ECG available during the hospitalization where the TdP event occurred. The methodology and validity of the QT measurement at KPNC can be found elsewhere.(Iribarren et al., 2013, 2014) In brief, all ECGs in the KPNC system are obtained using cardiographs manufactured by Philips Medical Systems (Andover, Massachusetts) which have a proprietary algorithm for QT measurement.(Kligfield et al., 2006) Bazett's correction for heart rate was used because is widely utilized in clinical practice.(Bazett, 1920) Hypokalemia was defined as a potassium level of lower than 3.6 millimoles per liter (mmol/L). Hypomagnesemia was defined as a serum magnesium level of lower than 1.5 milligrams per deciliter (mg/dl). Late‐stage chronic kidney disease was defined by an estimated glomerular filtration rate(Levey et al., 2009) of less than 30 milliliter per min (ml/min) or by codes for chronic dialysis. Active prescriptions for QT‐prolonging medications (as listed in *Crediblemeds*.*org*, accessed on 9/10/2020) as well as cholesterol‐lowering drugs prescribed within a 60‐day window prior to index hospitalization for TdP cases or index date for controls were identified utilizing the KPNC outpatient pharmacy database. Discharge disposition for cases and 30‐day, 6‐month, and one‐year mortality for both cases and controls were ascertained using complimentary validated sources of mortality in KPNC.(Arellano et al., 1984).

Additional variables were obtained through electronic chart review of unstructured fields in the EHR, including concomitant surgery, congenital long QT syndrome, left ventricular ejection fraction (EF) to characterize prior history of heart failure as heart failure with preserved ejection fraction (HFpEF; EF≥45%) or heart failure with reduced ejection fraction [HFrEF; EF<45%], and left ventricular hypertrophy (LVH; defined as at least 1.2 cm posterior wall or interventricular septal wall thickness). EF data from transthoracic echocardiograms (TTEs) were available in 32 TdP cases out of 33 and in 20 controls out of 21 with prior history of heart failure. LVH data were available from TTE performed within two years prior to the index hospitalization or index date for controls in 53 cases and 65 controls. Inpatient treatment for TdP management strategies, development of sustained ventricular fibrillation, cardiac arrest, and issuance of advanced directives and do not resuscitate (DNR) orders were determined via chart review. The study was approved by the Kaiser Foundation Research Institute Institutional Review Board.

### 2.2 Statistical analysis

Case–control differences were assessed using the t test for continuous variables and the chi‐square test for categorical variables. To determine independent predictors of TdP, we used conditional logistic regression with stepwise entry of covariates and a p value threshold to stay in the model of 0.05. To retain all subjects in the model, we created a dummy variable for missing LVH. All the statistical analyses were performed with SAS version 9.4. (SAS Institute Inc., North Carolina, USA).

## 3 RESULTS

Our TdP case validation over a 14‐year period provides a population‐based estimate of 3.6 cases of TdP per 100,000 persons per year. The 56 patients with confirmed TdP were, on average, 74 years old (*SD* = 13 years); 25 (45%) were men and 31 (55%) women. The majority of TdP cases were white (84%), and a small portion were black/African American (3.6%), Asian & Pacific Islander (3.6%), or mixed race (8.9%) (Table 1). There were no significant differences between cases and controls in body mass index, smoking status, or cholesterol‐lowering medication use. The baseline QT_c_ was 458 ± 40 ms in cases and 432 ± 35 ms in controls (*p *< .0001). Prolonged QTc (i.e., >480 ms) was present in 30 percent of cases and 10 percent of controls (*p *= .0008). The mean (SD) delta (event baseline) QTc in cases was 48 (53) ms. The average serum potassium level was lower in cases than in controls (3.7 vs. 4.3 mEq/L; *p *< .0001), and the proportion with low potassium (<3.6 mEq/L) was greater in cases than in controls (30 vs. 4.5%; *p *< .0001). Average magnesium concentrations did not significantly differ between TdP cases and controls, but the proportion with low magnesium was higher in cases than in controls (10 vs. 0%; *p *= .01).

**TABLE 1:** Baseline characteristics of TdP cases and controls

	TdP Cases *N* = 56	Controls *n* = 112	*p*
Age, years	73.6 ± 12.9	73.7 ± 12.7	0.98
Gender, (%)			
Female	31 (55.4%)	62 (55.4%)	1.00
Male	25 (44.6%)	50 (44.6%)	
Race, *n* (%)			
White	47 (83.9%)	94 (83.9%)	1.00
Black/African American	2 (3.6%)	4 (3.6%)	
Asian & Pacific Islander	2 (3.6%)	4 (3.6%)	
Mixed race	5 (8.9%)	10 (8.9%)	
Body mass index, Kg/m^2^	27.9 ± 5.9	28.8 ± 7.6	0.44
Smoking status, *n* (%)			
Current	4 (7.1%)	5 (4.5%)	0.55
Former	24 (42.9%)	57 (50.9%)	
Never	26 (46.4%)	43 (38.4%)	
Missing	2 (3.6%)	7 (6.3%)	
Cholesterol‐lowering medication, *n* 9%)	43 (76.8%)	78 (69.6%)	0.33
Baseline QTc, ms	458 ± 40	432 ± 35	<0.0001
Missing baseline QTc, *n* (%)	4 (7%)	0	
Baseline QTc ≥480 ms, *n* (%)	17 (30.4%)	11 (9.8%)	0.0008
Event QTc, ms	504 ± 54	NA	
∆ QTc, ms	48 ± 53	NA	
Potassium, mEq/L	3.7 ± 0.8	4.3 ± 0.5	<0.0001
Potassium <3.6 mEq/L, *n* (%)	17 (30.4%)	5 (4.5%)	<0.0001
Missing potassium, *n* (%)	2 (4%)	3 (3%)	
Magnesium, mEq/L	1. ± 0.6	2.0 ± 0.2	0.36
Magnesium <1.5 mg, *n* (%)	5 (10.0%)	0 (0)	0.01
Missing magnesium, *n* (%)	6 (11%)	53 (47%)	

Several prevalent comorbidities were found to differ between the two groups with a higher prevalence seen among the TdP cases, including the following: coronary artery disease (*p *= .003), HFpEF (*p *= .01), HFrEF (*p *= .0001), any cardiomyopathy (*p *< .0001), atrial fibrillation/flutter (*p *< .0001), left ventricular hypertrophy (*p *= .01), left bundle branch block (*p *= .0002), cardiac pacemaker (*p *= .02), automatic implantable cardioverter‐defibrillator (*p *= .0003), cardiac arrest (*p* < .0001), any valvular heart disease (*p *= .001), and late‐stage chronic kidney disease (*p *= .001) (Table 2). We also observed non‐significant trends for higher prevalence of diabetes, hypertension, stroke, HFpEF, bradycardia, pulmonary valve stenosis/regurgitation, and chronic liver disease among cases relative to controls.

**TABLE 2:** Prevalent comorbidities of TdP cases and controls

Comorbidities	TdP cases n=56	Controls n=112	*p*
Diabetes	18 (32.1%)	28 (25.0%)	.33
Hypertension	44 (78.6%)	72 (64.3%)	.06
Coronary artery disease	26 (46.4%)	27 (24.1%)	.003
Transient ischemic attack	8 (14.3%)	12 (10.7%)	.50
Ischemic stroke	8 (14.3%)	6 (5.4%)	.05
Hemorrhagic stroke	1 (1.8%)	1 (0.9%)	.62
Any cerebrovascular disease	11 (19.6%)	15 (13.4%)	.29
Heart failure with preserved ejection fraction (HFpEF)	16 (28.6%)	15 (13.4%)	.01
Heart failure with reduced ejection fraction (HFrEF)	16 (28.6%)	5 (4.5%)	<.0001
Heart failure with unknown ejection fraction	1 (1.7%)	1 (0.9%)	.80
Any heart failure	33 (58.9%)	21 (18.7%)	<.0001
Ischemic cardiomyopathy	9 (16.1%)	2 (1.8%)	.0004
Non‐ischemic cardiomyopathy	14 (25.0%)	5 (4.5%)	<.0001
Any cardiomyopathy	16 (28.6%)	6 (5.4%)	<.0001
Bradycardia	5 (8.9%)	5 (4.5%)	.25
Atrial fibrillation/flutter	34 (60.7%)	32 (28.6%)	<.0001
Paroxysmal supraventricular tachycardia	3 (5.4%)	5 (4.5%)	.79
Left ventricular hypertrophy (LVH)	16 (28.5%)	14 (12.5%)	.01
Unknown left ventricular hypertrophy (LVH)	3 (5.3%)	47 (41.9%)	<.0001
Left bundle branch block	11 (19.6%)	3 (2.7%)	.0002
Right bundle branch block	2 (3.6%)	4 (3.6%)	1.0000
Any bundle branch block	13 (23.2%)	7 (6.3%)	.001
Cardiac pacemaker	10 (17.9%)	7 (6.3%)	.02
Automatic implantable cardioverter‐defibrillator (AICD)	8 (14.3%)	1 (0.9%)	.0003
Cardiac arrest	19 (33.9%)	2 (1.8%)	<.0001
Mitral valve stenosis/regurgitation	14 (25.0%)	10 (8.9%)	.005
Aortic valve stenosis/regurgitation	13 (23.2%)	10 (8.9%)	.01
Tricuspid valve stenosis/regurgitation	11 (19.6%)	3 (2.7%)	.0002
Pulmonary valve stenosis/regurgitation	0 (0.0%)	1 (0.9%)	.48
Any heart valve disease	23 (41.1%)	20 (17.9%)	.001
Late‐stage chronic kidney disease	28 (50.0%)	28 (25.0%)	.001
Chronic liver disease	5 (8.9%)	5 (4.5%)	.25

The ten most common QT‐prolonging prescriptions administered to TdP cases within 60 days of index hospitalization and overall percentages of “other” and “any” QT‐prolonging medications (along with corresponding prescription utilization in controls also within 60 days prior to their index date) are shown in Table 3. TdP cases were significantly more likely to have been prescribed furosemide (17.7% vs. 6.5%; *p *= .007), amiodarone (7.3% vs. 0.0%; *p *= .001), and “any” QT‐prolonging drugs (80.2% vs. 57.6%; *p *= .0003). On the other hand, controls were more likely to have been prescribed “other” (i.e., other than the 10 most common among cases) QT‐prolonging drugs (43.8% vs. 69.8%; *p *< .0001). The full list of QT‐prolonging drugs is provided as (online‐only) Appendix SB.

**TABLE 3:** QT‐prolonging drugs* in TdP cases and controls

Drug	TdP Cases (*n* = 56)	Controls (*n* = 112)	*p*
Furosemide	17 (17.7%)	9 (6.5%)	.007
Hydrochlorothiazide	8 (8.3%)	6 (4.3%)	.20
Omeprazole	8 (8.3%)	10 (7.2%)	.75
Amiodarone	7 (7.3%)	0 (0.0%)	.001
Famotidine	3 (3.1%)	7 (5.0%)	.47
Mirtazapine	3 (3.1%)	1 (0.7%)	0.16
Escitalopram	2 (2.1%)	0 (0.0%)	.08
Hydrocodone Bitartrate/Acetaminophen	2 (2.1%)	8 (5.8%)	.17
Sotalol	2 (2.1%)	1 (0.7%)	.35
Torsemide	2 (2.1%)	0 (0.0%)	.08
Other	42 (43.8%)	97 (69.8%)	<.0001
Any	77 (80.2%)	80 (57.6%)	.0003

*10 most common in cases.

Stepwise conditional logistic regression analysis was performed to determine independent risk factors for TdP development within our 56 cases compared with the 112 matched controls. Statistically significant risk factors included the following: potassium concentration <3.6 mEq/L (OR = 10.6; *p *< .0001), atrial fibrillation/flutter (OR = 6.2; *p *< .0001), QTc >480 ms (OR = 4.4; *p *= .01), and prior history of coronary artery disease (OR = 2.6; *p *= .03) (Table 4).

**TABLE 4:** Independent predictors of TdP by stepwise conditional logistic regression in the full sample of cases (*n* = 56) and controls (*n* = 112)

Independent Predictors	OR (95% CI)	*p*
Potassium <3.6 mEq/L	10.6 (2.54–43.9)	<.0001
Atrial fibrillation/flutter	6.25 (2.13–18.3)	<.0001
QTc >480 ms	4.38 (1.19–16.1)	.01
Coronary artery disease	2.59 (1.06–6.34)	.03

TdP resulted in cardiac arrest in 44 patients (78.6%) and sustained ventricular fibrillation in 31 (55.4%) of patients. None of the cases had been admitted for surgical procedures, and two cases (3.6%) and no controls (0%) had a prior diagnosis of congenital long QT syndrome.

The most frequent treatment for TdP was intravenous magnesium sulfate (78.6%) and intravenous potassium chloride (73.2%). Anti‐arrhythmic drug therapies, including lidocaine (48.2%) and amiodarone (35.7%), were utilized less commonly. Overdrive pacing internally (transvenous pacemaker or permanent pacemaker) or externally (intravenous dopamine or isoproterenol) was utilized in 12 out of the 56 (21.4%) TdP patients. A total of 17 out of the 51 (33.3%) patients underwent cardiopulmonary resuscitation with defibrillation. Of note, five patients had an active do not resuscitate (DNR) directive within their medical record.

Six out of 56 TdP patients (10.7%) died during the hospitalization. The underlying cause of death was aspiration pneumonia (*n* = 2), cardiogenic shock (*n* = 1), severe sepsis (*n* = 1), ventricular tachycardia (*n* = 1), and myocarditis (*n* = 1). Eight died within 1 year after discharge, yielding a cumulative mortality of 25.0% at 1 year. After hospital discharge, 1 death occurred within 30 days, 1 death between 30 days and 6 months, and 6 deaths between 6 months and 1 year. All controls were alive one year after their “index date” hospitalization.

## 4 DISCUSSION

Our study confirms that TdP is a rare occurrence (3.6 cases per 100,000 per year) with serious consequences: 55 percent developed sustained ventricular fibrillation, 11 percent died in the hospital, and 17 percent died within 12 months of discharge. Our rate (which was derived from a population‐based cohort) is lower than the incidence reported in a Belgian study (34 per 100,000 per year) that was based on patients admitted to a tertiary hospital in a 3‐year period(Vandael et al., 2017a) but higher than the incidence of drug‐induced LQTS/TdP in Berlin (0.25 per 100,000 per year in males and 0.40 per 100,000 per year in females) determined by active surveillance of 51 hospitals between 2008 and 2011.(Sarganas et al., 2014).

The independent risk factors for TdP included, in order of strength of association, hypokalemia (i.e., potassium concentration <3.6 mEq/L), prior history of atrial fibrillation/flutter, prolonged QTc (>480 ms), and prior history of coronary artery disease. Other risk factors that were significantly different between TdP cases and controls in a univariate analysis did not emerge as statistically significant predictors in the multivariate context. This could be the result of limited statistical power and the fact that these variables were often highly correlated with each other.

Certain electrophysiological events are known to be associated with the development of TdP; drugs which prolong the action potential duration (APD), induce early afterdepolarizations and ectopic beats, and increase the dispersion of ventricular repolarization are more likely to result in increased risk of arrhythmia and TdP development. Prolongation of the ventricular APD by inhibition of the I_Kr_ current is the primary mechanism of Class III anti‐arrhythmic agents (i.e., dofetilide, amiodarone, and sotalol), which are often used in management of arrhythmias in heart failure patients. However, with prolongation of the APD and QT interval, these treatments are known to be arrhythmogenic, with increased risk of TdP development.(Belardinelli et al., 2003) Some clinical trials have suggested congestive heart failure to elevate the risk of TdP due to higher use of anti‐arrhythmic therapy with blocking of the I_Kr_ current, as stated above.(Roden et al., 2007) Low extracellular potassium paradoxically reduces I_Kr_ by enhanced inactivation or exaggerated competitive block by sodium leading to QT prolongation and dispersion of repolarization; prompt correction of extracellular potassium levels can quickly shorten the QT interval and associated morphologic abnormalities.(Kallergis et al., 2012) This literature is in line with our results showing exposure to two QT‐prolonging drugs occurred significantly more frequently in TdP cases than in controls: amiodarone (a Class III anti‐arrhythmic) and furosemide (a loop diuretic). The association of diuretic use with in‐hospital TdP can be explained by its correlation with congestive heart failure and electrolyte derangements, such as hypokalemia. Our results are also in line with case reports linking TdP with cardiomyopathy (Kurisu et al., 2008) and renal disease.(Patane et al., 2008).

In a scientific statement from the American Heart Association and the American College of Cardiology Foundation, Drew et al. state a gradual increase in QTc interval increases the risk of TdP development with each 10‐ms increase in QTc leading to a 5%–7% exponential increase in the risk for TdP.(Drew et al., 2010) Within our cohort, we observed an average increase of 48 ms in QTc between the baseline and the event QT among the TdP cases. This absolute increase in QTc helps to identify patients who should have continuous ECG monitoring. At present, no quantitative multivariate risk index exists for the prediction of TdP in the hospital‐based population. Several risk factors have been postulated based on pathophysiologic mechanisms and small case series; however, our real‐world and largest case–control study was able to uniquely identify several independent risk predictors which can guide practitioners on which patients may qualify for continuous QTc or ECG monitoring and/or electrolyte replacement therapy.

Similar to the case series by Kay et al, we also found pre‐existing structural heart disease, arrhythmias, hypokalemia, and QT‐prolonging medications to be commonly seen in patients who develop TdP.(Kay et al., 1983) Kay et al. found quinidine to be the most common QT‐prolonging medication; however, in recent years the use of quinidine has substantially reduced and other QT‐prolonging medications such as amiodarone and furosemide are more commonly seen. Kay et al. stated the effectiveness of drug treatment was unpredictable and unreliable since so few cases had been reported.

The most common treatment utilized for TdP was intravenous magnesium. Most patients also received IV potassium repletion, regardless of potassium levels. One‐third of patients underwent defibrillation and about one‐fifth of patients were internally or externally overdrive paced. Overdrive pacing by a transvenous pacemaker, increasing the baseline permanent pacemaker rate, or increasing the heart rate by pharmacologic means (intravenous dopamine or isoproterenol) was used in hopes of shortening the QT interval and terminating TdP. Given the known QT‐prolonging side effects of amiodarone, anti‐arrhythmic treatment with intravenous amiodarone was used less commonly.

**FIGURE 1:**
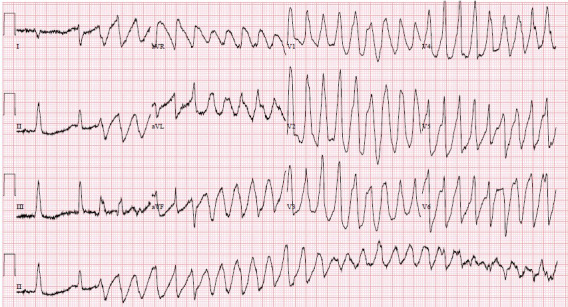
Illustrative example of a 12‐lead ECG of a patient presenting with TdP

We highlight one example from our case series describing the development and management of TdP. He is an 82‐year‐old man with history of coronary artery bypass grafting, dual‐chamber permanent pacemaker for complete heart block, ischemic cardiomyopathy with a left ventricular ejection fraction of 45%, and prostate cancer on chronic ketoconazole therapy who presented with syncope at home. Upon arrival to the emergency department, he suffered recurrent loss of consciousness and 12‐lead ECG was performed demonstrating polymorphic ventricular tachycardia (Figure 1). He was immediately treated with initiation of intravenous lidocaine, intravenous magnesium sulfate, and intravenous potassium chloride. All QT‐prolonging medications, including his chronic ketoconazole treatment, were stopped. He was successfully treated by increasing the lower rate limit on his dual‐chamber permanent pacemaker to shorten the QT interval and prevent future TdP. He was discharged from the hospital alive with no recurrent polymorphic ventricular tachycardia.

We recognize several limitations in our case–control study. First, our sample size was small given the rare nature of TdP, limiting statistical power. Second, although we leveraged a real‐world population with ethnic diversity, all patients included were insured, and thus, our results may not generalize to uninsured populations. Third, we did not consider genetic data since this is part of a separate effort and is beyond the scope of this manuscript. Fourth, we did not develop RISQ‐PATH scores because of missing hs‐CRP values.(Vandael, Vandenberk, Vandenberghe, Spriet, et al., 2017).

TdP is an uncommon but potentially fatal arrhythmia due to selective potential of action potential durations in certain layers of the ventricular myocardium leading to dispersion of repolarization and a prolonged QT interval. In light of the recent COVID‐19 pandemic and the use of off‐label treatment strategies for SARS‐CoV‐2 which are known to prolong QTc, there is heightened importance of identifying risk predictors for TdP development reported showing intravenous potassium and magnesium repletion and overdrive pacing to be the most commonly utilized strategies.

Further research is needed to understand the combined effects of predisposing genetic makeup, medication effects, and clinical risk factors. Thereafter, appropriate mitigation and management strategies can be fully understood.

## CONFLICT OF INTEREST

The authors have no conflicts to disclose.

## ETHICAL APPROVAL

This study was approved by the Kaiser Foundation Research Institute Institutional Review Board and the requirement for informed consent was waived.

## AUTHOR CONTRIBUTIONS

Neha Mantri, Wrote the manuscript, approved final version. Meng Lu, Statistical analysis, approved final version. Jonathan G. Zaroff, Critical review, approved final version. Neil Risch, Critical review, approved final version. Thomas Hoffmann, Critical review, approved final version. Akinyemi Oni‐Orisan, Critical review, approved final version. Catherine Lee, Statistical methods, approved final version. Carlos Iribarren, Cowrote the manuscript, approved final version.

## Supporting information

Supplementary Material

Supplementary Material

## Data Availability

No individual data are available, but authors would contemplate reasonable requests.
