# Supplementary material for: Torsade de pointes: A nested case–control study in an integrated healthcare delivery system
Source: Ann Noninvasive Electrocardiol. 2021 Sep 21;27(1):e12888. doi: 10.1111/anec.12888 (PMC8739596; doi:10.1111/anec.12888)
Supplement: Supplementary file 1 — Supplementary Material [file ANEC-27-e12888-s001.docx]

**Appendix A.** ICD-9, ICD-10 and CPT4 codes for ascertainment of comorbidities.

| Comorbidities | ICD-9 | ICD-10 | CPT4 |
| --- | --- | --- | --- |
| Hypertension | 401, 401.1, 401.9  405.9, 405.91  405.99, 996.81 | I10 – I16 |  |
| Coronary artery disease | 411.1, 413.1, 413.9, 410, 412, 36.01, 36.02,  36.03, 36.05, 36.06, 36.07, 36.09, 36.10, 36.11, 36.12, 36.13, 36.14, 36.15, 36.16, 36.17, 36.19, 36.03, 36.2, 36.3, 00.66, 295.5, V45.82 | 0210*, 0211*, 0212*, 0213*, 02703*, 02713*, 02723*, 02733*, Z95.5, Z98.61 | 92980, 92981, 92982, 92984, 92995, 92996, 92975, 92977, 33510, 33511, 92920, 92921, 92924, 92925, 92928, 92929, 92933, 92934, 92937, 92938, 92941, 92943, 92944, 33510-33536 |
| Ischemic stroke | 433.01, 433.11, 433.21, 433.31, 433.81, 433.91, 434.01, 434.11, 434.91, 437.0, 437.1 | I63, I67.89, I69.320, I69.398 |  |
| Hemorrhagic stroke | 430, 431, 432.1, 432.9 | I60, I61, I62 |  |
| Heart failure | 428.**, 402.01, 402.11, 402.91, 398.91, 404.01, 404.03, 404.11 | I50.**, I11.0, I09.81, I13.0, I13.2, I13.11 |  |
| Ischemic cardiomyopathy | 414.8 | I25.5 |  |
| Non-ischemic cardiomyopathy | 425.0, 425,1, 425.11, 425.18, 425.2, 425.3, 425.4, 425.5, 425.7,425.8, 425.9 | I42.9, I43 |  |
| Bradycardia | 427.81 | R00.1 |  |
| Atrial fibrillation / flutter | 427.31, 427.32 | I48.0, I48.1, I48.2, I48.91, I48.92 |  |
| Paroxysmal supraventricular tachycardia | 427.0 | I47.1 |  |
| Left bundle branch block | 426.3 | I44.7 |  |
| Right bundle branch block | 426.4 | I44.10 |  |
| Cardiac pacemaker | V45.01, V53.31, 295.0 | Z45, Z95.0 | 33217, 33218, 33220, 33221, 33222,33223, 33224, 33225, 33226, 33227,33228, 33229, 33230, 33231, 33233,33234, 33235, 33236, 33236, 33237,33238, 33240, 33244, 33249, 33262,33263, 33264, 33270, 33271, 33272,33273 |
| Automatic implantable cardioverter defibrillator (AICD) | V45.02 | Z45.02, Z95.810 | 33249 |
| Cardiac arrest | 427.5 | I46,2, I46.8, I46.9 |  |
| Mitral valve stenosis/regurgitation | 394.0, 394.1, 394.2, 396.2,  396.9, 424.0 | I34.1, I05.1, I34.0, I08.0  I34.0 - I34.9, I05.0 |  |
| Aortic valve stenosis/regurgitation | 424.1, 396.3, 395.0, 396.0, 746.3 | I35.9, I06.1, I35.1, I35.2,  I35.0, I06.2 |  |
| Tricuspid valve stenosis/regurgitation | 397.0, 746.89, 093.23, 746.1, 424.2 | I36.1, I36.0 |  |
| Pulmonary valve stenosis/regurgitation | 424.3, 746.02 | I37.1, I37.0 |  |
| Late-stage chronic kidney disease | 585.4, 585.5 | N18.4, N18.5 | 90935, 90936, 90937, 90938, 90939, 90940, 90941, 90942, 90943, 90944, 90945, 90946, 90947, 90951, 90952, 90953, 90954, 90955, 90956, 90957, 90958, 90959, 90960, 90961, 90962, 90963, 90964, 90965, 90966, 90967, 90968, 90969, 90970, 99070, 99289, 99290, 99512, 99559 |
| Chronic liver disease | 571.x, 572.8 | K70-K77 |  |

All codes are inpatient (primary discharge diagnosis), except for hypertension (outpatient codes).
