# Supplementary material for: Torsade de pointes: A nested case–control study in an integrated healthcare delivery system
Source: Ann Noninvasive Electrocardiol. 2021 Sep 21;27(1):e12888. doi: 10.1111/anec.12888 (PMC8739596; doi:10.1111/anec.12888)
Supplement: Supplementary file 2 — Supplementary Material [file ANEC-27-e12888-s002.docx]

| **Generic Name** | **Drug Class** |
| --- | --- |
| Abarelix (Only on Non US Market) | GnRH Antagonist |
| Abiraterone | Anti-androgen |
| Aclarubicin (Only on Non US Market) | Anti-cancer |
| Alfuzosin | Alpha-1 adrenergic blocker |
| Alimemazine (Trimeprazine) (Only on Non US Market) | Antihistamine |
| Amantadine | Antiviral |
| Amiodarone | Antiarrhythmic |
| Amisulpride | Antiemetic, Antipsychotic |
| Amitriptyline | Antidepressant, Tricyclic |
| Amphotericin B | Antifungal |
| Amsacrine (Acridinyl anisidide) (Only on Non US Market) | Antineoplastic Agent |
| Anagrelide | Phosphodiesterase 3 inhibitor |
| Apalutamide | Nonsteroidal antiandrogen |
| Apomorphine | Dopamine agonist |
| Aripiprazole | Antipsychotic, atypical |
| Arsenic trioxide | Anti-cancer |
| Artemether/Lumefantrine | Anti-malarial |
| Artenimol/piperaquine (Only on Non US Market) | Antimalarial |
| Asenapine | Antipsychotic, atypical |
| Astemizole (Removed from US Market) | Antihistamine |
| Atazanavir | Antiviral |
| Atomoxetine | CNS stimulant |
| Azithromycin | Antibiotic |
| Bedaquiline | Antibiotic |
| Bendamustine | Anti-cancer |
| Bendroflumethiazide (Bendrofluazide) | Diuretic, thiazide |
| Benperidol (Only on Non US Market) | Antipsychotic |
| Bepridil | Antianginal |
| Betrixaban | Anticoagulant |
| Bortezomib | Proteasome inhibitor |
| Bosutinib | Anti-cancer |
| Buprenorphine | Opioid agonist |
| Cabozantinib | Anti-cancer |
| Capecitabine | Anti-cancer |
| Carbetocin (Only on Non US Market) | Uterotonic |
| Ceritinib | Anti-cancer |
| Cesium Chloride | Toxin |
| Chloral hydrate | Sedative |
| Chloroquine | Antimalarial |
| Chlorpromazine | Antipsychotic / Antiemetic |
| Chlorprothixene (Only on Non US Market) | Antipsychotic |
| Cilostazol | Phosphodiesterase 3 inhibitor |
| Cimetidine | Antacid |
| Ciprofloxacin | Antibiotic |
| Cisapride (Removed from US Market) | GI stimulant |
| Citalopram | Antidepressant, SSRI |

k

| Clarithromycin | Antibiotic |
| --- | --- |
| Clofazimine (Only on Non US Market) | Antibiotic |
| Clomipramine | Antidepressant, Tricyclic |
| Clotiapine (Only on Non US Market) | Antipsychotic, atypical |
| Clozapine | Antipsychotic, atypical |
| Cobimetinib | Anti-cancer |
| Cocaine | Local anesthetic |
| Crizotinib | Anti-cancer |
| Cyamemazine (Cyamepromazine) (Only on Non US Mar | etAntipsychotic |
| Dabrafenib | Anti-cancer |
| Dasatinib | Anti-cancer |
| Degarelix | Anti-androgen |
| Delamanid (Only on Non US Market) | Antibiotic |
| Desipramine | Antidepressant, Tricyclic |
| Deutetrabenazine | Vesicular monamine transporter 2 inhibitor |
| Dexmedetomidine | Sedative |
| Dextromethorphan/Quinidine | Unknown |
| Diphenhydramine | Antihistamine |
| Disopyramide | Antiarrhythmic |
| Dofetilide | Antiarrhythmic |
| Dolasetron | Antiemetic |
| Domperidone (Only on Non US Market) | Antiemetic |
| Donepezil | Cholinesterase inhibitor |
| Doxepin | Antidepressant, Tricyclic |
| Dronedarone | Antiarrhythmic |
| Droperidol | Antipsychotic / Antiemetic |
| Efavirenz | Antiviral |
| Eliglustat | Glucosylceramide synthase inhibitor |
| Encorafenib | BRAF inhibitor |
| Entrectinib | Anti-cancer |
| Eperisone (Only on Non US Market) | Antispasmodic |
| Epirubicin | Anti-cancer |
| Eribulin mesylate | Anti-cancer |
| Erythromycin | Antibiotic |
| Escitalopram | Antidepressant, SSRI |
| Esomeprazole | Proton Pump Inhibitor |
| Ezogabine (Retigabine) | Anticonvulsant |
| Famotidine | H2-receptor antagonist |
| Felbamate | Anticonvulsant |
| Fingolimod | Sphingosine phospate receptor modulator |
| Flecainide | Antiarrhythmic |
| Fluconazole | Antifungal |
| Fluorouracil (5-FU) | Anti-cancer |
| Fluoxetine | Antidepressant, SSRI |
| Flupentixol (Only on Non US Market) | Antipsychotic |
| Fluvoxamine | Selective Serotonin Reuptake Inhibitor |
| Furosemide (frusemide) | Diuretic |

| Galantamine | Cholinesterase inhibitor |
| --- | --- |
| Garenoxacin (Only on Non US Market) | Antibiotic |
| Gatifloxacin (Removed from US Market) | Antibiotic |
| Gemifloxacin | Antibiotic |
| Gilteritinib | Antineoplastic |
| Glasdegib | Anti-cancer |
| Granisetron | Antiemetic |
| Grepafloxacin (Removed from US Market) | Antibiotic |
| Halofantrine (Only on Non US Market) | Antimalarial |
| Haloperidol | Antipsychotic |
| Hydrochlorothiazide | Diuretic |
| Hydrocodone - ER | Analgesic |
| Hydroquinidine (Dihydroquinidine) (Only on Non US Mark | Antiarrhythmic |
| Hydroxychloroquine | Antimalarial, Anti-inflammatory |
| Hydroxyzine | Antihistamine |
| Ibogaine (Only on Non US Market) | Psychedelic |
| Ibutilide | Antiarrhythmic |
| Iloperidone | Antipsychotic, atypical |
| Imipramine (Melipramine) | Antidepressant, Tricyclic |
| Indapamide | Diuretic |
| Inotuzumab ozogamicin | Anti-cancer |
| Isradipine | Antihypertensive |
| Itraconazole | Antifungal |
| Ivabradine | Antianginal |
| Ivosidenib | IDH1 inhibitor |
| Ketanserin (Only on Non US Market) | Antihypertensive |
| Ketoconazole | Antifungal |
| Lacidipine (Only on Non US Market) | Calcium channel blocker |
| Lansoprazole | Proton Pump Inhibitor |
| Lapatinib | Anti-cancer |
| Lefamulin | Antibiotic |
| Lenvatinib | Anti-cancer |
| Leuprolide (Leuprorelin) | Anti-androgen |
| Levofloxacin | Antibiotic |
| Levomepromazine (Methotrimeprazine) (Only on Non US | Antipsychotic |
| Levomethadone (levamethadone) (Only on Non US Marke | Opioid |
| Levomethadyl acetate (Removed from US Market) | Opioid agonist |
| Levosulpiride (Only on Non US Market) | Antipsychotic |
| Lithium | Antimanic |
| Lofexidine | Alpha-2-adrenergic agonist, central |
| Loperamide | Opioid agonist |
| Lopinavir/Ritonavir | Antiviral |
| Lumateperone | Antipsychotic, atypical |
| Lurasidone | Antipsychotic, atypical |
| Maprotiline | Anti-depressant, Tetracyclic |
| Melperone (Only on Non US Market) | Antipsychotic, atypical |
| Memantine | NMDA receptor antagonist |

| Mesoridazine (Removed from US Market) | Antipsychotic |
| --- | --- |
| Methadone | Opioid agonist |
| Metoclopramide | Antiemetic |
| Metolazone | Diuretic |
| Metronidazole | Antibiotic |
| Mianserin (Only on Non US Market) | Anti-depressant |
| Midostaurin | Anti-cancer |
| Mifepristone | Progesterone antagonist |
| Mirabegron | Beta3 adrenergic antagonist |
| Mirtazapine | Antidepressant, Tetracyclic |
| Moexipril/Hydrochlorothiazide | Antihypertensive |
| Moxifloxacin | Antibiotic |
| Necitumumab | Anti-cancer |
| Nelfinavir | Antiviral |
| Nicardipine | Antihypertensive |
| Nifekalant (Only on Non US Market) | Antiarrhythmic |
| Nilotinib | Anti-cancer |
| Norfloxacin | Antibiotic |
| Nortriptyline | Antidepressant, Tricyclic |
| Nusinersen | Antisense oligonucleotide |
| Ofloxacin | Antibiotic |
| Olanzapine | Antipsychotic, atypical |
| Omeprazole | Proton Pump Inhibitor |
| Ondansetron | Antiemetic |
| Osilodrostat | Aldosterone synthase inhibitor |
| Osimertinib | Anti-cancer |
| Oxaliplatin | Anti-cancer |
| Oxytocin | Oxytocic |
| Paliperidone | Antipsychotic, atypical |
| Palonosetron | Antiemetic |
| Panobinostat | Histone deacetylase inhibitor |
| Pantoprazole | Proton Pump Inhibitor |
| Papaverine HCl (Intra-coronary) | Vasodilator, Coronary |
| Paroxetine | Antidepressant, SSRI |
| Pasireotide | Somatostatin analog |
| Pazopanib | Anti-cancer |
| Pentamidine | Antifungal |
| Perflutren lipid microspheres | Imaging contrast agent |
| Perphenazine | Antipsychotic |
| Pilsicainide (Only on Non US Market) | Anti-arrhythmic |
| Pimavanserin | Antipsychotic, atypical |
| Pimozide | Antipsychotic |
| Pipamperone (Only on Non US Market) | Antipsychotic |
| Piperacillin/Tazobactam | Antibiotic |
| Pitolisant (Tiprolisant) | Histamine 3 antagonist/inverse agonist |
| Posaconazole | Antifungal |
| Pretomanid | Antitubercular |

| Primaquine phosphate | Antimalarial |
| --- | --- |
| Probucol (Removed from US Market) | Antilipemic |
| Procainamide | Antiarrhythmic |
| Promethazine | Antipsychotic / Antiemetic |
| Propafenone | Sodium channel blocker |
| Propofol | Anesthetic, general |
| Prothipendyl (Only on Non US Market) | Antipsychotic |
| Quetiapine | Antipsychotic, atypical |
| Quinidine | Antiarrhythmic |
| Quinine sulfate | Antimalarial |
| Ranolazine | Antianginal |
| Ribociclib | Anti-cancer |
| Rilpivirine | Antiviral |
| Risperidone | Antipsychotic, atypical |
| Romidepsin | Histone deacetylase inhibitor |
| Roxithromycin (Only on Non US Market) | Antibiotic |
| Rucaparib | PARP inhibitor |
| Saquinavir | Antiviral |
| Selpercatinib | Kinase inhibitor |
| Sertindole (Only on Non US Market) | Antipsychotic, atypical |
| Sertraline | Antidepressant, SSRI |
| Sevoflurane | Anesthetic, general |
| Siponimod |  |
| Solifenacin | Muscle relaxant |
| Sorafenib | Anti-cancer |
| Sotalol | Antiarrhythmic |
| Sparfloxacin (Removed from US Market) | Antibiotic |
| Sulpiride (Only on Non US Market) | Antipsychotic, atypical |
| Sultopride (Only on Non US Market) | Antipsychotic, atypical |
| Sunitinib | Anti-cancer |
| Tacrolimus | Immunosuppressant |
| Tamoxifen | Anti-cancer |
| Tazemetostat | Anti-cancer |
| Telaprevir | Antiviral |
| Telavancin | Antibiotic |
| Telithromycin | Antibiotic |
| Terfenadine (Removed from US Market) | Antihistamine |
| Terlipressin (Only on Non US Market) | Vasoconstrictor |
| Terodiline (Only on Non US Market) | Muscle relaxant |
| Tetrabenazine | Vesicular Monoamine Transporter 2 Inhibitor |
| Thioridazine | Antipsychotic |
| Tiapride (Only on Non US Market) | Selective D2, D3 dopamine antagonist |
| Tipiracil/Trifluridine | Anti-cancer |
| Tizanidine | Muscle relaxant |
| Tolterodine | Muscle relaxant |
| Toremifene | Estrogen agonist/antagonist |
| Torsemide (Torasemide) | Diuretic |

| Tramadol | Analgesic |
| --- | --- |
| Trazodone | Antidepressant, SARI |
| Trimipramine | Antidepressant, Tricyclic |
| Tropisetron (Only on Non US Market) | Antiemetic |
| Valbenazine | Vesicular monamine transporter 2 inhibitor |
| Vandetanib | Anti-cancer |
| Vardenafil | Phosphodiesterase 5 inhibitor |
| Vemurafenib | Anti-cancer |
| Venlafaxine | Antidepressant, SNRI |
| Voriconazole | Antifungal |
| Vorinostat | Histone deacetylase inhibitor |
| Ziprasidone | Antipsychotic, atypical |
| Zotepine (Only on Non US Market) | Antipsychotic, atypical |
| Zuclopenthixol (Zuclopentixol) (Only on Non US Market) | Antipsychotic |
